# Increased Susceptibility to Ethylmercury-Induced Mitochondrial Dysfunction in a Subset of Autism Lymphoblastoid Cell Lines

**DOI:** 10.1155/2015/573701

**Published:** 2015-01-21

**Authors:** Shannon Rose, Rebecca Wynne, Richard E. Frye, Stepan Melnyk, S. Jill James

**Affiliations:** Department of Pediatrics, University of Arkansas for Medical Sciences, Arkansas Children's Hospital Research Institute, 13 Children's Way, Slot 512-41B, Little Rock, AR 72202, USA

## Abstract

The association of autism spectrum disorders with oxidative stress, redox imbalance, and mitochondrial dysfunction has become increasingly recognized. In this study, extracellular flux analysis was used to compare mitochondrial respiration in lymphoblastoid cell lines (LCLs) from individuals with autism and unaffected controls exposed to ethylmercury, an environmental toxin known to deplete glutathione and induce oxidative stress and mitochondrial dysfunction. We also tested whether pretreating the autism LCLs with N-acetyl cysteine (NAC) to increase glutathione concentrations conferred protection from ethylmercury. Examination of 16 autism/control LCL pairs revealed that a subgroup (31%) of autism LCLs exhibited a greater reduction in ATP-linked respiration, maximal respiratory capacity, and reserve capacity when exposed to ethylmercury, compared to control LCLs. These respiratory parameters were significantly elevated at baseline in the ethylmercury-sensitive autism subgroup as compared to control LCLs. NAC pretreatment of the sensitive subgroup reduced (normalized) baseline respiratory parameters and blunted the exaggerated ethylmercury-induced reserve capacity depletion. These findings suggest that the epidemiological link between environmental mercury exposure and an increased risk of developing autism may be mediated through mitochondrial dysfunction and support the notion that a subset of individuals with autism may be vulnerable to environmental influences with detrimental effects on development through mitochondrial dysfunction.

## 1. Introduction

Autism spectrum disorders (ASD) are defined as a heterogeneous group of neurodevelopmental disorders characterized by impairments in communication and social interactions along with restrictive and repetitive behaviors [1]. The incidence of ASD in the United States is currently estimated to be 1 in 68 individuals, and it continues to rise [2]. While the etiology of ASD remains unknown, multiple interacting genetic and environmental factors are thought to contribute to the development of ASD. In addition to behavioral impairments, recent studies indicate that many children with ASD also exhibit impairments in energy production and redox homeostasis [3–5].

Multiple studies have demonstrated the presence of glutathione-mediated redox imbalance and oxidative stress in individuals with ASD [5–11]. Our group has consistently reported decreased concentrations of glutathione (GSH) and several of its metabolic precursors as well as increased oxidized glutathione disulfide (GSSG) and a decreased glutathione redox ratio (GSH/GSSG) in plasma, immune cells, and postmortem brain from children with ASD [4, 5, 11–13]. Oxidative stress and damage have been documented in blood and brain of individuals with ASD including reports of decreased levels and activities of antioxidant enzymes and elevated levels of oxidized lipids, proteins, and DNA [4, 7, 8, 11, 14, 15]. In primary lymphocytes and in lymphoblastoid cell lines (LCLs) derived from children with autistic disorder (AD), we have found that the production of reactive oxygen species (ROS) is elevated as compared to controls [12, 13, 16]. The imbalance between glutathione-mediated antioxidant capacity and ROS production in autism LCLs may cause these cells to be more susceptible to oxidative stress and damage from any exogenous sources of ROS as compared to control LCLs.

Recent evidence indicates that the incidence of mitochondrial dysfunction in ASD may be very high, affecting up to 30% or more of children with ASD [17]. While the etiology of mitochondrial dysfunction in ASD is not known, evidence suggests that oxidative stress may be a key factor driving mitochondrial dysfunction in individuals with ASD [16, 18]. Recently, we demonstrated that LCLs derived from children with AD exhibit abnormal mitochondrial respiration at baseline as well as a more rapid decline in mitochondrial function upon exposure to increasing ROS as compared to LCLs from control children [16]. Importantly, we found that these abnormal mitochondrial parameters were driven by a subgroup consisting of 32% of the AD LCLs (termed AD-A for abnormal), whereas the other autism LCLs (termed AD-N for normal) had mitochondrial parameters similar to controls. Furthermore, we also demonstrated that pretreatment of the AD LCLs with N-acetyl cysteine (NAC) increased intracellular GSH and the GSH/GSSG redox ratio and, in the AD-A subgroup, conferred protection from mitochondrial dysfunction when ROS was increased [18].

Mitochondria are both the primary producers and targets of intracellular ROS due to the continuous low-level production of superoxide that accompanies electron transfer across the inner mitochondrial membrane during oxidative phosphorylation [19]. ROS are also generated from other sources such as activated immune cells [3, 17] and prooxidant environmental toxicants such as pesticides, diesel exhaust, and mercury [20–29]. Mercury is one of several environmental toxicants that have been found to have an association with the development of ASD [28, 30–34]. Ethylmercury is an established a sulfhydryl reagent that rapidly binds to and depletes intracellular glutathione and increases intracellular ROS in a dose-dependent manner [12, 35]. We have previously demonstrated that AD LCLs have increased susceptibility to oxidative stress from exposure to ethylmercury such that exposure to ethylmercury resulted in lower intracellular GSH and GSH/GSSG and increased ROS production in AD LCLs as compared to control LCLs [12].

In the present study we tested the hypothesis that the subset of AD LCLs previously found to exhibit mitochondrial dysfunction when challenged with ROS would also exhibit mitochondrial dysfunction when exposed to ethylmercury (i.e., ethylmercury-induced mitochondrial dysfunction). Furthermore, we hypothesized that pretreatment with NAC to increase the intracellular glutathione concentration would confer protection from ethylmercury-induced mitochondrial dysfunction. To this end, we used extracellular flux analysis to measure mitochondrial oxygen consumption in AD and control LCLs transiently exposed to ethylmercury. The AD LCLs were also tested after pretreatment with NAC to determine whether changes in mitochondrial bioenergetics after exposure to ethylmercury could be prevented by NAC-induced increase in intracellular glutathione-mediated redox capacity.

## 2. Methods

### 2.1. Lymphoblastoid Cell Lines and Culture Conditions

Sixteen LCLs derived from white males diagnosed with AD chosen from pedigrees with at least 1 affected male sibling (mean/SD age 7.9 ± 2.6 years) were obtained from the Autism Genetic Resource Exchange (Los Angeles, CA, USA) or the National Institutes of Mental Health (NIMH; Bethesda, MD, USA) center for collaborative genomic studies on mental disorders.  Table 1 denotes the AD LCL subgroups previously classified: five were classified as AD-A (for abnormal) and the remaining eleven were classified as AD-N (for normal) [16]. Sixteen control LCLs derived from healthy white male donors with no documented behavioral or neurological disorder or first-degree relative with a medical disorder that could involve abnormal mitochondrial function (mean/SD age 19.3 ± 11.5 y) were obtained from Coriell Cell Repository (Camden, NJ, USA) or the NIMH. On average, cells were studied at passage 12, with a maximum passage of 15. Genomic stability is very high at this low passage number [36, 37]. Cells were maintained in RPMI 1640 culture medium with 15% FBS and 1% penicillin/streptomycin (Invitrogen, Grand Island, NY, USA) in a humidified incubator at 37°C with 5% CO_2_.

### 2.2. Mitochondrial Respiratory Function Assay

**Figure 1:**
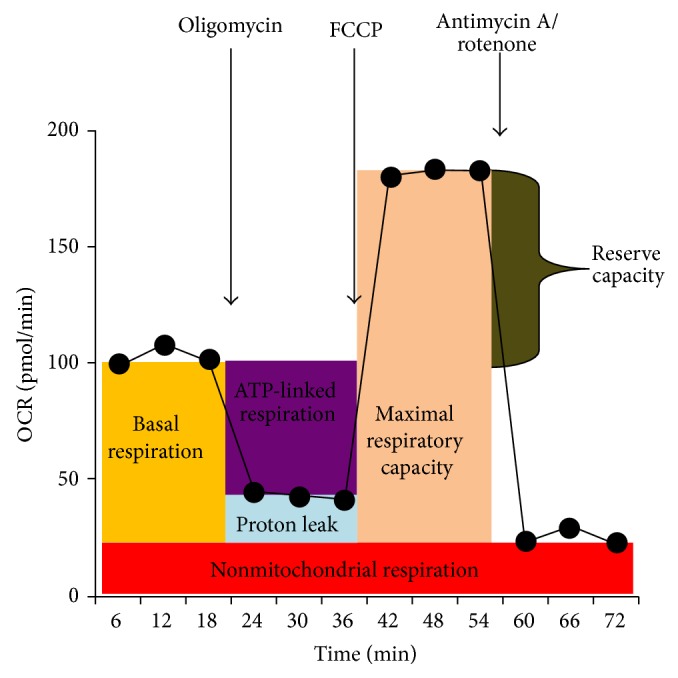
Assay of mitochondrial respiratory function. Several parameters of mitochondrial respiration are derived by measuring the oxygen consumption rate (OCR) upon the sequential addition of mitochondrial inhibitors. Basal OCR is first measured, from which nonmitochondrial respiration is subtracted to derive basal respiration. Oligomycin, a complex V inhibitor, is used to derive ATP-linked respiration (basal OCR minus oligomycin OCR) and proton leak respiration (oligomycin OCR minus nonmitochondrial respiration). Carbonyl cyanide-p-trifluoromethoxyphenylhydrazone (FCCP), a protonophore, collapses the inner membrane proton gradient, allowing the ETC to function at its maximal rate, and maximal respiratory capacity can be derived (FCCP OCR minus nonmitochondrial respiration). Antimycin A and rotenone, inhibitors of complexes III and I, inhibit all ETC function, revealing the nonmitochondrial respiration. Reserve capacity is derived from the maximal and basal rates (maximal OCR minus basal OCR).

We used extracellular flux analysis (Seahorse Bioscience, Inc., North Billerica, MA, USA) to measure the oxygen consumption rate (OCR), an indicator of mitochondrial respiration, in real-time in live intact LCLs as described [16]. Upon the sequential addition of mitochondrial electron transport chain (ETC) inhibitors and an uncoupler to the respiring cells, several parameters of mitochondrial respiration were derived, including basal respiration, ATP-linked respiration, proton leak respiration, and reserve capacity (diagramed in Figure 1). Briefly, after measuring basal respiration, oligomycin, an inhibitor of complex V, is added, and the resulting OCR is used to derive ATP-linked respiration and proton leak respiration. Next carbonyl cyanide-p-trifluoromethoxyphenylhydrazone (FCCP), a protonophore, is added to collapse the inner membrane gradient, allowing the ETC to function to its maximal rate, and maximal respiratory capacity is derived. Lastly, antimycin A, a complex III inhibitor, and rotenone, a complex I inhibitor, are added to shut down ETC function, revealing the nonmitochondrial respiration, which is subtracted from the other rates to obtain the corrected basal respiration, proton leak respiration and maximal respiratory capacity. The mitochondrial reserve capacity is calculated by subtracting basal respiration from maximal respiratory capacity.

Two hours prior to the assay, cells were seeded onto poly-D-lysine coated 96-well XF-PS plates at a density of 1.1 × 10^5^ cells/well in DMEM XF assay media (unbuffered DMEM supplemented with 11 mM glucose, 2 mM L-glutamax, and 1 mM sodium pyruvate). Cells were plated with at least 3 replicate wells for each treatment group. Optimal concentrations of oligomycin (1.0 *μ*M), FCCP (0.3 *μ*M), antimycin A (0.3 *μ*M), and rotenone (1.0 *μ*M) were carefully titrated.

### 2.3. Ethylmercury Challenge

Cells were exposed to increasing concentrations of ethylmercury for 2 h prior to the mitochondrial assay. Thimerosal (Sigma-Aldrich, St. Louis, MO, USA), a mercurial compound composed of 49.6% ethylmercury by weight, was diluted in DMEM XF assay media into 10X stocks and added to cells in an XF-PS plate and incubated for 2 h at 37°C in a non-CO_2_ incubator. The final concentrations of ethylmercury were 0.063 *μ*M, 0.125 *μ*M, 0.25 *μ*M and 0.5 *μ*M, 1.25 *μ*M, and 2.5 *μ*M.

### 2.4. Pretreatment with N-Acetyl Cysteine

**Figure 2:**
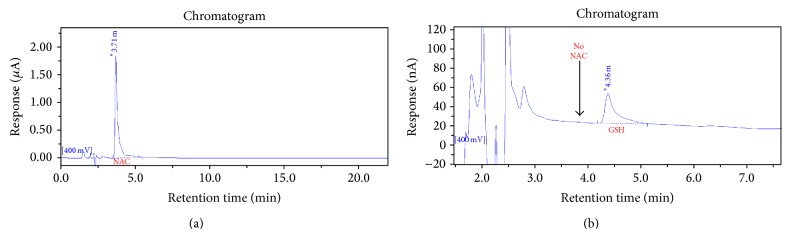
No residual NAC remains in pretreated cells following washing regimen. HPLC chromatograms from a standard preparation of NAC (a) and two million AD LCLs pretreated with NAC and washed two times with DMEM XF assay media (b). The NAC peak seen at retention time 3.71 min in the standard preparation (a) is not detectable in the cell extract, whereas the GSH peak at 4.36 min is prominent (b).

To determine whether pretreatment with a glutathione precursor could rescue any atypical response to the ROS challenge, AD LCLs were plated in T25 flasks at a density of 5.0 × 10^5^ cells/mL in culture media with or without 1 mM NAC for 48 h prior to the assay. Control LCLs were cultured identically without NAC. Cells were washed twice in DMEM XF media to remove any remaining NAC prior to mercury treatment and mitochondrial assays. To confirm this regimen was sufficient to remove all NAC from the cells, 2.0 × 10^6^ NAC-pretreated cells were pelleted following the two washes and analyzed by HPLC, as previously described, for the presence of NAC [12]. Presented in Figure 2 is a representative chromatogram which demonstrates that there was no NAC remaining in the NAC-pretreated cells following the two washes.

### 2.5. Analytic Approach

A mixed-model regression [38] was conducted via SAS version 9.3 (Cary, NC, USA) “Glimmix” Procedure. The mixed-model allowed data from each AD LCL to be compared to the paired control LCL run on the same plate. The mitochondrial respiratory parameter was the response variable with a between-group dichotomous effect (e.g., AD versus control) and within-group repeated factor of ethylmercury concentration (modeled as a multilevel factor) as well as the interaction between these effects. We present the* overall* difference between the two comparison groups (group effect), the overall effect of the ethylmercury concentration (ethylmercury effect), and whether the effect of ethylmercury concentration was different between the two groups (ethylmercury x group interaction). The same analysis was used to analyze the difference in mitochondrial respiratory parameters between each AD subgroup and matched controls. We then analyzed the effect of pretreatment with NAC on the AD LCLs. This effect is analyzed for each AD subgroup separately. For all models, random effects included the intercept and ethylmercury. *F*-tests were used to evaluate significance. Planned post hoc orthogonal contrasts were used when the interaction was significant.

## 3. Results

### 3.1. Mitochondrial Function in AD LCLs with Ethylmercury Challenge

**Figure 3:**
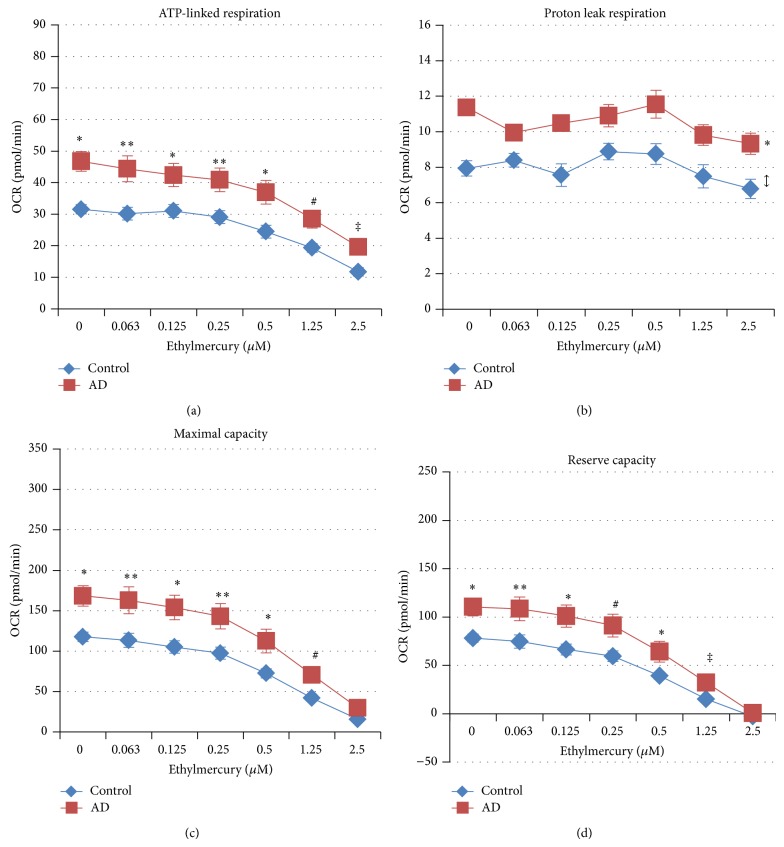
AD LCLs demonstrate abnormal mitochondrial function at baseline and following ethylmercury exposure. (a) ATP-linked respiration, (b) proton leak respiration, (c) maximal respiratory capacity, and (d) reserve capacity are all overall significantly higher in the AD LCLs as compared to control LCLs. ATP-linked respiration is higher in the AD LCLs at every concentration of ethylmercury, while maximal respiratory capacity and reserve capacity are higher in the AD LCLs at every concentration except the highest concentration. ^*^ *P* ≤ 0.0001; ^**^ *P* ≤ 0.001; ^#^ *P* ≤ 0.01; ^‡^ *P* ≤ 0.05; *↕* indicates an overall statistical difference between LCL groups when differences at individual concentrations are not significant.

ATP-linked respiration was overall higher for AD LCLs as compared to control LCLs [*F*(1, 662) = 134.55, *P* < 0.0001] (Figure 3(a)). ATP-linked respiration decreased significantly as ethylmercury concentration increased [*F*(4, 89) = 30.95, *P* < 0.0001] and was found to be significantly lower than baseline at 0.5 *μ*M [*t*(89) = 3.90, *P* < 0.001], 1.25 *μ*M [*t*(89) = 6.94, *P* < 0.0001] and 2.5 *μ*M [*t*(89) = 11.53, *P* < 0.0001] ethylmercury in both groups. However, the decrease in ATP-linked respiration with increasing ethylmercury concentration was significantly different between the AD and control groups [*F*(6, 662) = 2.42, *P* < 0.05]. The differences in ATP-linked respiration between the two LCL groups were significant at every concentration of ethylmercury [0 *μ*M *t*(662) = 9.51; 0.063 *μ*M *t*(662) = 4.33; 0.125 *μ*M *t*(662) = 4.30; 0.25 *μ*M *t*(662) = 3.59; 0.5 *μ*M *t*(662) = 4.57; 1.25 *μ*M *t*(662) = 3.20; 2.5 *μ*M *t*(662) = 2.91]. However, the difference was smaller at high ethylmercury concentrations because the drop in ATP-linked respiration was greater for the AD LCLs as compared to the control LCLs as ethylmercury concentration increased.

Proton leak respiration was overall higher in AD LCLs as compared to the control LCLs [*F*(1, 662) = 136.09, *P* < 0.0001] (Figure 3(b)). Proton leak respiration changed significantly in both groups as ethylmercury concentration increased [*F*(4, 89) = 2.55, *P* < 0.05] with a significantly lower proton leak respiration at 2.5 *μ*M ethylmercury as compared to baseline [*t*(89) = 2.64, *P* < 0.01], but this change was not different between the two groups.

Maximal respiratory capacity was overall higher for AD LCLs as compared to control LCLs [*F*(1, 662) = 100.89, *P* < 0.0001] (Figure 3(c)). Maximal respiratory capacity decreased significantly as ethylmercury concentration increased [*F*(4, 89) = 35.90, *P* < 0.0001] and was found to be significantly lower than baseline at 0.5 *μ*M [*t*(89) = 4.55, *P* < 0.001], 1.25 *μ*M [*t*(89) = 7.88, *P* < 0.0001], and 2.5 *μ*M [*t*(89) = 11.40, *P* < 0.0001] ethylmercury in both AD and control LCLs. However, the decrease in maximal respiratory capacity with increasing ethylmercury concentration was significantly different between the AD and control groups [*F*(6, 662) = 3.08, *P* < 0.01]. Maximal respiratory capacity was significantly higher in the AD LCLs at each concentration of ethylmercury except the highest concentration [0 *μ*M *t*(662) = 8.62; 0.063 *μ*M *t*(662) = 3.75; 0.125 *μ*M *t*(662) = 4.66; 0.25 *μ*M *t*(662) = 3.44; 0.5 *μ*M *t*(662) = 3.84; 1.25 *μ*M *t*(662) = 2.46]. The decrease in maximal respiratory capacity was greater for the AD LCLs as compared to the control LCLs as ethylmercury concentration increased.

Reserve capacity was overall higher for AD LCLs as compared to control LCLs [*F*(1, 662) = 76.77, *P* < 0.0001] (Figure 3(d)). Reserve capacity decreased significantly as ethylmercury concentration increased [*F*(4, 89) = 33.54, *P* < 0.0001] and was found to be significantly lower than baseline at 0.5 *μ*M [*t*(89) = 4.56, *P* < 0.001], 1.25 *μ*M [*t*(89) = 7.66, *P* < 0.0001], and 2.5 *μ*M [*t*(89) = 10.68, *P* < 0.0001] ethylmercury in both AD and control LCLs. However, the decrease in reserve capacity with increasing ethylmercury concentration was significantly different between the AD and control groups [*F*(6, 662) = 3.38, *P* < 0.01]. Reserve capacity was significantly higher in the AD LCLs at each concentration of ethylmercury except the highest concentration [0 *μ*M *t*(662) = 7.81; 0.063 *μ*M *t*(662) = 3.39; 0.125 *μ*M *t*(662) = 4.50; 0.25 *μ*M *t*(662) = 3.17; 0.5 *μ*M *t*(662) = 3.32; 1.25 *μ*M *t*(662) = 1.93]. The decrease in reserve capacity was greater for the AD LCLs as compared to the control LCLs as ethylmercury concentration increased.

### 3.2. Mitochondrial Function in AD LCLs Subgroups with Ethylmercury Challenge

To better understand the differences between the two AD LCL subgroups we previously identified, we compared the AD LCLs to their paired control LCLs within each subgroup. There were no significant differences found between the two subsets of control LCLs matched to the subsets of AD LCLs.

#### 3.2.1. AD-A versus Control LCLs

**Figure 4:**
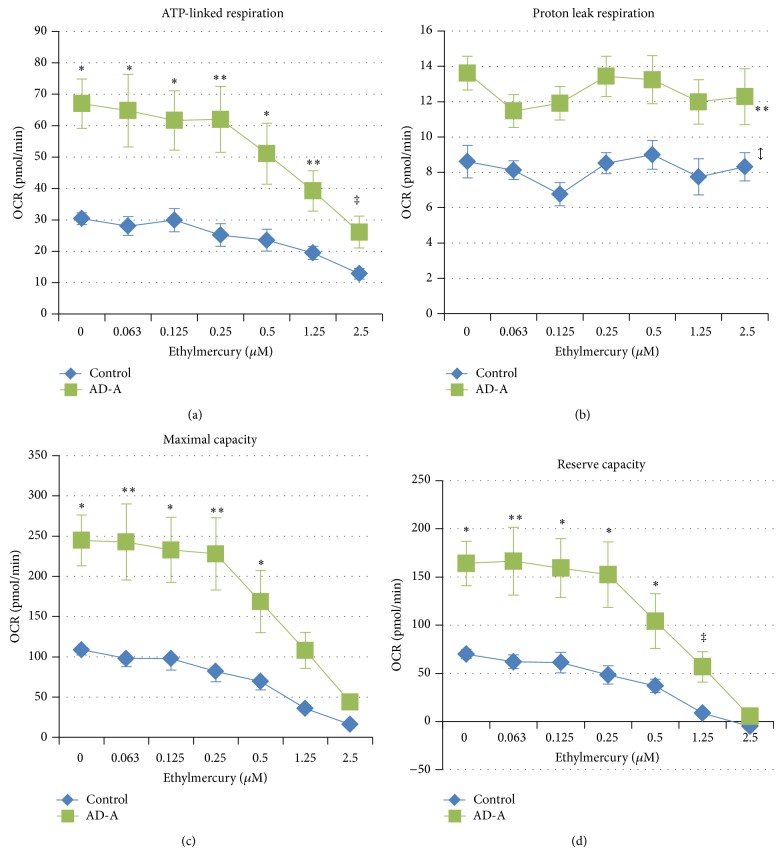
Distinct mitochondrial respiratory parameters and responses to ethylmercury in the AD-A LCL subgroup. (a) ATP-linked respiration is significantly higher in the AD-A LCLs as compared to paired control LCLs at every concentration. (b) Proton leak respiration is overall markedly higher in the AD-A LCLs as compared to paired control LCLs. (c) Maximal respiratory capacity and (d) reserve capacity are both significantly higher in AD-A LCLs as compared to paired control LCLs at every concentration of ethylmercury except the highest concentration. ^*^ *P* ≤ 0.0001; ^**^ *P* ≤ 0.001; ^#^ *P* ≤ 0.01; ^‡^ *P* ≤ 0.05; *↕* indicates an overall statistical difference between LCL groups when differences at individual concentrations are not significant.

ATP-linked respiration was markedly higher for AD-A LCLs as compared to control LCLs [*F*(1, 213) = 183.13, *P* < 0.0001] (Figure 4(a)). ATP-linked respiration decreased significantly as ethylmercury concentration increased [*F*(6, 24) = 9.11, *P* < 0.0001] and was found to be significantly lower than baseline at 1.25 *μ*M [*t*(24) = 2.92, *P* < 0.01] and 2.5 *μ*M [*t*(24) = 5.65, *P* < 0.0001] ethylmercury in both AD-A and control LCLs. However, the decrease in ATP-linked respiration with increasing ethylmercury concentration was significantly different between the two LCL groups [*F*(6, 213) = 3.95, *P* < 0.001]. Although the differences in ATP-linked respiration between the two LCL groups were significant at each concentration of ethylmercury [0 *μ*M *t*(213) = 10.97; 0.063 *μ*M *t*(213) = 5.63; 0.125 *μ*M *t*(213) = 5.60; 0.25 *μ*M *t*(213) = 4.90; 0.5 *μ*M *t*(213) = 5.03; 1.25 *μ*M *t*(213) = 3.62; 2.5 *μ*M *t*(213) = 2.45], the difference between the LCL groups was reduced at high ethylmercury concentrations because the drop in ATP-linked respiration was greater for the AD-A LCLs as compared to the control LCLs as ethylmercury increased.

Overall, proton leak respiration was markedly higher for AD-A LCLs [*F*(1, 213) = 124.56, *P* < 0.001] (Figure 4(b)). Proton leak respiration did not significantly change as ethylmercury concentration increased nor was there any difference in the change in proton leak respiration with increasing ethylmercury concentration between the AD-A and control LCL groups.

Maximal respiratory capacity was markedly higher for AD-A LCLs as compared to control LCLs [*F*(1, 213) = 148.63, *P* < 0.0001] (Figure 4(c)). Maximal respiratory capacity decreased significantly as the ethylmercury concentration increased [*F*(6, 24) = 8.09, *P* < 0.0001] and was found to be significantly lower than baseline at 1.25 *μ*M [*t*(24) = 3.12, *P* < 0.01] and 2.5 *μ*M [*t*(24) = 4.94, *P* < 0.0001] ethylmercury in both AD-A and control LCLs. However, the decrease in maximal respiratory capacity with increasing ethylmercury concentration was significantly different between the two LCL groups [*F*(6, 213) = 5.00, *P* < 0.0001]. The differences in maximal respiratory capacity between the two LCL groups were significant at each concentration of ethylmercury except the highest concentration [0 *μ*M *t*(213) = 10.32, *P* < 0.0001; 0.063 *μ*M *t*(213) = 5.20, *P* < 0.001; 0.125 *μ*M *t*(213) = 5.82, *P* < 0.0001; 0.25 *μ*M *t*(213) = 4.54, *P* < 0.001; 0.5 *μ*M *t*(213) = 4.33, *P* < 0.0001; 1.25 *μ*M *t*(213) = 2.66, *P* = 0.01]. This demonstrates that there was a greater drop in maximal respiratory capacity for the AD-A LCLs as compared to the control LCLs as ethylmercury concentration increased.

Reserve capacity was markedly higher for AD-A LCLs as compared to control LCLs [*F*(1, 213) = 123.94, *P* < 0.0001] (Figure 4(d)). Reserve capacity decreased significantly as ethylmercury concentration increased [*F*(6, 24) = 7.72, *P* = 0.0001] and was found to be significantly lower than baseline at 1.25 *μ*M [*t*(24) = 3.11, *P* < 0.01] and 2.5 *μ*M [*t*(24) = 4.73, *P* < 0.0001] ethylmercury in both AD-A and control LCLs. However, the decrease in reserve capacity with increasing ethylmercury concentration was significantly different between the two LCL groups [*F*(6, 213) = 5.48, *P* < 0.0001]. The differences in reserve capacity between the two LCL groups were significant at each concentration of ethylmercury except the highest concentration [0 *μ*M *t*(213) = 9.78; 0.063 *μ*M *t*(213) = 4.93; 0.125 *μ*M *t*(213) = 5.64; 0.25 *μ*M *t*(213) = 4.20; 0.5 *μ*M *t*(213) = 3.89; 1.25 *μ*M *t*(213) = 2.21]. This demonstrates that there was a greater drop in reserve capacity for the AD-A LCLs as compared to the control LCLs as ethylmercury concentration increased.

#### 3.2.2. AD-N versus Control LCLs

As the focus of this study is on the abnormal AD-A subgroup, the detailed results for the AD-N subgroup are presented in the Supplementary Files (see Supplementary Files in Supplementary Material available online at http://dx.doi.org/10.1155/2015/573701). Briefly, ATP-linked respiration [*F*(1, 442) = 18.96, *P* < 0.0001], proton leak respiration [*F*(1, 442) = 38.16, *P* < 0.0001], and maximal respiratory capacity [*F*(1, 442) = 7.13, *P* < 0.01] were all overall slightly, but significantly, higher in the AD-N subgroup as compared to control LCLs, while there was no difference in reserve capacity between the AD-N and control LCLs (Supplementary Figure S1). The magnitude of the differences between the AD-N and control LCLs is much less than those observed between the AD-A and control LCLs, and importantly, the mitochondrial response to increasing concentrations of ethylmercury was not different between the AD-N and control LCLs.

### 3.3. The Effect of NAC Pretreatment

We examined the effects of pretreating the AD LCLs for 48 hours with NAC on baseline mitochondrial respiration as well as the change in mitochondrial respiration following ethylmercury exposure. We examined these effects on the two AD LCL subgroups separately.

#### 3.3.1. AD-A LCLs: NAC Pretreatment versus No Pretreatment

**Figure 5:**
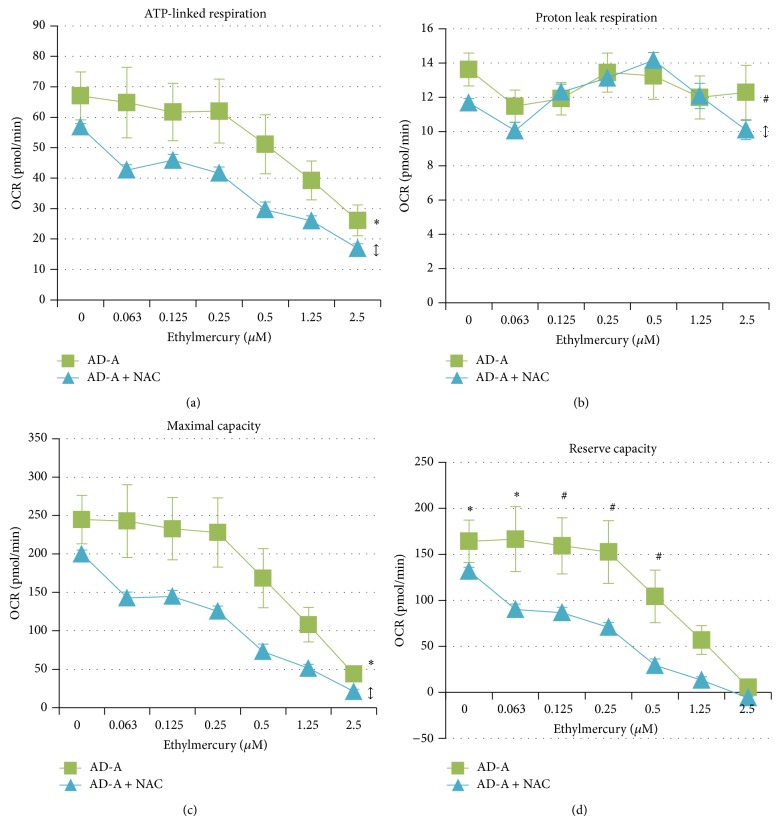
Mitochondrial respiratory parameters and responses to ethylmercury differ in the AD-A LCL subgroup following 48-hour pretreatment with 1 mM N acetyl-cysteine (NAC). (a) ATP-linked respiration, (c) maximal respiratory capacity, and (d) reserve capacity are overall markedly significantly lower in the AD-A LCLs pretreated with NAC as compared to the nonpretreated group. (b) Proton leak respiration is only slightly but significantly lower in the NAC pretreated group as compared to the nonpretreated group. (d) Reserve capacity is markedly lower in the NAC pretreated AD-A LCLs at every concentration except the two highest concentrations. Furthermore, the drop in reserve capacity is significantly blunted in the AD-A LCLs pretreated with NAC as compared to the nonpretreated group. ^*^ *P* ≤ 0.0001; ^#^ *P* ≤ 0.01; *↕* indicates an overall statistical difference between LCL groups when differences at individual concentrations are not significant.

Pretreatment with NAC markedly reduced ATP-linked respiration in the AD-A LCLs [*F*(1, 212) = 33.60, *P* < 0.0001] (Figure 5(a)). ATP-linked respiration for both the NAC pretreated and the nonpretreated AD-A LCLs decreased as ethylmercury concentration increased [*F*(6, 24) = 8.30, *P* < 0.0001], but this decrease was not different between the two groups. ATP-linked respiration was significantly lower than baseline at 1.25 *μ*M [*t*(24) = 3.72, *P* = 0.001] and 2.5 *μ*M [*t*(24) = 5.87, *P* < 0.0001] ethylmercury in both NAC-pretreated and nonpretreated AD-A LCLs.

Pretreatment with NAC slightly but significantly decreased proton leak respiration in the AD-A LCLs [*F*(1, 212) = 5.62, *P* = 0.01] (Figure 5(b)). Overall proton leak for both the NAC pretreated and the nonpretreated AD-A LCLs did not change as ethylmercury concentration was increased and there was no difference in the change in proton leak respiration with the increase in ethylmercury.

Pretreatment with NAC markedly decreased maximal respiratory capacity in the AD-A LCLs [*F*(1, 212) = 40.86, *P* < 0.0001] (Figure 5(c)). Maximal respiratory capacity for both the NAC pretreated and the nonpretreated AD-A LCLs decreased as ethylmercury concentration increased [*F*(6, 24) = 8.36, *P* < 0.0001], but this decrease was not different between the two groups. Maximal respiratory capacity was significantly lower than baseline at 1.25 *μ*M [*t*(24) = 3.84, *P* < 0.001] and 2.5 *μ*M [*t*(24) = 5.55, *P* < 0.0001] ethylmercury in both NAC-pretreated and nonpretreated AD-A LCLs.

Pretreatment with NAC markedly decreased overall reserve capacity in the AD-A LCLs [*F*(1, 212) = 43.40, *P* < 0.001] (Figure 5(d)). Reserve capacity for both the NAC pretreated and the nonpretreated AD-A LCLs decreased as ethylmercury concentration increased [*F*(6, 24) = 7.72, *P* = 0.0001] and was significantly lower than baseline at 1.25 *μ*M [*t*(24) = 3.72, *P* = 0.001] and 2.5 *μ*M [*t*(24) = 5.23, *P* < 0.0001] ethylmercury in both groups. This decrease in reserve capacity was different between the two LCL groups [*F*(6, 212) = 2.14, *P* = 0.05]. Importantly, the differences in reserve capacity between the two LCL groups were significant at each concentration of ethylmercury except the two highest concentrations demonstrating that there was a greater drop in reserve capacity for the AD-A LCLs which were not pretreated with NAC as compared to the NAC pretreated AD-A LCLs as ethylmercury increased [0 *μ*M *t*(212) = 5.31; 0.063 *μ*M *t*(212) = 3.96; 0.125 *μ*M *t*(212) = 3.03; 0.25 *μ*M *t*(212) = 2.71; 0.5 *μ*M *t*(212) = 2.61].

#### 3.3.2. AD-N LCLs: NAC Pretreatment versus No Pretreatment

The detailed results of NAC pretreatment on the AD-N LCL subgroup are presented in the Supplementary Files. Briefly, NAC pretreatment resulted in a slight but significant increase in ATP-linked respiration [*F*(1, 505) = 23.00, *P* < 0.0001], proton leak respiration [*F*(1, 505) = 10.74, *P* = 0.001], and maximal respiratory capacity [*F*(1, 505) = 5.20, *P* < 0.05] in AD-N LCLs at baseline. Importantly, the change in mitochondrial parameters with ethylmercury exposure was not different in AD-N LCLs with NAC pretreatment as compared to AD-N LCLs without pretreatment (Supplementary Figure S2).

## 4. Discussion

This study examined mitochondrial respiration in lymphoblastoid cell lines (LCLs) derived from children with autism at baseline and following exposure to the environmental toxin, ethylmercury, and the protective potential of NAC pretreatment. We show that LCLs derived from children with autism exhibit significant abnormalities in mitochondrial respiration at baseline with these abnormalities worsening following exposure to ethylmercury. At baseline, AD LCLs exhibit what appeared to be overactive mitochondria as evidenced by higher ATP-linked and proton leak respiration, maximal respiratory capacity, and reserve capacity. Following exposure to increasing concentrations of ethylmercury, we demonstrate a greater decrease in ATP-linked respiration as well as maximal respiratory and reserve capacity in the AD LCLs as compared to the control LCLs. These findings were driven by the abnormal (AD-A) subset of AD LCLs, which exhibit markedly abnormal mitochondrial parameters and have previously been shown to exhibit increased sensitivity to ROS, resulting in ROS-induced mitochondrial dysfunction [16]. Pretreatment of the AD-A subgroup with NAC significantly decreased the abnormally high mitochondrial parameters at baseline and blunted the loss of reserve capacity in response to ethylmercury. Overall, this study supports the notion that a subset of children with AD may have significant inherent physiological abnormalities in mitochondrial function and an increased vulnerability to oxidative environmental toxicants such as ethylmercury, which can induce mitochondrial dysfunction. The study also indicates that NAC may mitigate mitochondrial dysfunction and attenuate the effects of ethylmercury.

We previously classified our AD LCLs as normal (AD-N) or abnormal (AD-A) based on reserve capacity at baseline and the change in response to increasing ROS using 2,3-dimethoxy-1,4-naphthoquinone (DMNQ), an agent which produces hydrogen peroxide and superoxide upon entering a cell [16]. In the present study using ethylmercury as a prooxidant environmental stressor, the AD-A LCLs again exhibit a greater depletion of reserve capacity following ethylmercury exposure, as compared to control LCLs, despite having increased reserve capacity at baseline. Reserve capacity is a measure of the ability of the cell to increase mitochondrial oxidative phosphorylation to meet an increased ATP demand, and the increased reserve capacity at baseline in the AD-A subgroup is likely representative of an abnormal adaptive mitochondrial response as the AD-N subgroup does not exhibit an increased reserve capacity at baseline. However, the AD-A LCLs are unable to maintain the apparent adaptive increase in reserve capacity under conditions of acute oxidative stress. Rapid loss of reserve capacity following ethylmercury exposure is significant as loss of reserve capacity has been associated with several disease states including aging, heart disease and neurodegenerative diseases [39–42], and complete depletion of reserve capacity has been shown to result in cell death [42–45].

In addition to reserve capacity, ATP-linked respiration and maximal respiratory capacity are also significantly elevated in the AD-A subgroup at baseline. ATP-linked respiration is the portion of the electron transport chain (ETC) function that produces ATP. The remainder of ETC function is measured as proton leak, a mechanism used to regulate oxidative stress at the inner mitochondrial membrane. Maximal respiratory capacity is a measure of the maximal ability of the electron transport chain (ETC) to produce energy. The increase in ATP-linked respiration and maximal respiratory capacity indicates increased activity of the electron transport chain (ETC), which supports the notion of an increased ATP demand and compensatory overactivity of the ETC in the AD-A LCLs which we have demonstrated previously [16, 18], and is consistent with reported ETC overactivity in children with ASD [46, 47]. The AD-A LCLs are unable to maintain these elevated mitochondrial activities following ethylmercury exposure, exhibiting a greater drop in ATP-linked respiration and maximal respiratory capacity than in control cells. This causes the sharp decrease in reserve capacity. These data indicate that despite a background of increased ROS production and decreased glutathione-mediated redox capacity, the AD-A LCLs are capable of maintaining ATP production under basal conditions; however, they are more vulnerable to mitochondrial dysfunction when stressed with ethylmercury-induced oxidative stress.

Overall proton leak respiration is significantly higher in AD-A LCLs as compared to control LCLs, a finding that is consistent with increased ROS production and overall increased mitochondrial activity in the AD-A LCLs. Proton leak is a mechanism used by the cell to reduce ETC ROS generation by reducing mitochondrial membrane potential [48]. One of the major mechanisms to increase proton leak during conditions of chronic oxidative stress is the upregulation of uncoupling protein 2 (UCP2) [49–51], and we previously have shown increased UCP2 protein levels in the AD-A LCLs as compared to the AD-N LCLs [16]. The effect of increasing ethylmercury on proton leak was overall quite small compared to the effects on ATP-linked and maximal oxygen consumption. While ethylmercury can lead to increased ROS production in the LCLs [12], our data indicate that, at the exposures examined, the primary effect of ethylmercury on mitochondrial function is reducing ATP-linked and maximal respiration, likely by direct damage to the ETC complexes, rather than increasing proton leak. Iron-sulfur clusters are very sensitive to inactivation by mercury, and indeed methylmercury has been shown to directly damage several complexes in the ETC [52, 53].

Pretreatment with NAC provides the cells with cysteine, the rate-limiting amino acid for GSH synthesis. A 48 h pretreatment of the AD LCLs was used to allow sufficient time for cysteine deacetylation and incorporation into GSH. While intracellular glutathione content was not evaluated in this study, we have previously demonstrated that pretreatment of the AD LCLs with the same dose of NAC for 48 h results in significantly increased glutathione levels and redox status (GSH/GSSG) [18]. Pretreatment of the AD-A LCL subgroup with NAC results in a significant reduction in ATP-linked and proton leak respiration as well as maximal and reserve capacity, and it significantly blunts the loss of reserve capacity following ethylmercury exposure. NAC pretreatment may increase cellular GSH content and thus antioxidant capacity, leading to a reduction in the abnormally high ATP-linked respiration and maximal and reserve capacity and an improved ability to maintain reserve capacity during ethylmercury exposure. In the context of ethylmercury exposure, increased cellular GSH content from NAC pretreatment may also chelate ethylmercury and reduce the cellular ethylmercury concentration, resulting in an improved mitochondrial reserve capacity.

Interestingly, NAC pretreatment does not similarly affect the AD-N LCLs but instead results in slightly increased ATP-linked respiration, proton leak respiration, and maximal capacity, while having no effect on reserve capacity (see Supplementary Figure S2). We have previously described how the baseline mitochondrial parameters of the AD-A LCLs likely represent a maladaptive mitochondrial response that is characterized by markedly elevated ATP-linked and proton leak respiration and maximal and reserve capacity [16]. On the other hand, the AD-N LCLs represent a normal adaptive response that is characterized by only slight changes in mitochondrial respiration including slight increases in ATP-linked and proton leak respiration. It is possible in this normal adaptive situation that adding NAC relieves a relatively mild oxidative stress burden and actually boosts mitochondrial function, while in the maladaptive AD-A LCLs it relieves a more serious oxidative stress burden, lessening the ATP demand and reducing mitochondrial overactivity.

We demonstrate that NAC pretreatment does confer some protection from the ethylmercury-induced loss of reserve capacity in the AD-A LCLs, providing preliminary in vitro support for the clinical use of NAC to treat oxidative stress in autism. NAC can protect against oxidative stress-induced mitochondrial dysfunction [18, 54–56] as well as mitochondrial-generated oxidative stress [57]. In a mouse model of complex 1 deficiency, NAC was shown to improve cognitive deficits [58]. Importantly, a double-blind trial of NAC in children with autism proved efficacious in reducing symptoms of irritability suggesting that reduced glutathione redox capacity and oxidative stress may also contribute to behavioral symptoms associated with autism [59].

In the present study the mitochondrial respiratory response to mercury-induced oxidative stress was examined in AD and control LCLs using the ethylmercury-containing compound, thimerosal. Thimerosal has been shown to deplete glutathione and increase ROS [12, 35], to induce DNA strand breaks, membrane permeability and apoptosis, and to be cytotoxic at nanomolar and micromolar concentrations [60–63]. Evidence suggests that thimerosal induces apoptosis through a mitochondrial pathway [62, 64, 65] and that it is toxic to the mitochondria, reducing mitochondrial respiration and inducing mitochondrial DNA damage and superoxide production [65, 66]. Until recently thimerosal was used as a preservative in vaccines and pharmaceuticals with some vaccines containing 12.5 *μ*g to 25 *μ*g of ethylmercury per 0.5 mL dose, equating to approximately 100–200 *μ*M [67]. While the concentrations of ethylmercury used in this study (0.0625 *μ*M to 2.5 *μ*M) are at least 2 orders of magnitude lower than those once used in vaccines, any extrapolation of the dose response characteristics of this in vitro LCL model to the in vivo situation would be overstating and unsubstantiated. While ethylmercury was chosen in this study as an example of a prooxidant environmental toxin, another common environmental exposure to mercury is methylmercury in the diet. The toxicities of methylmercury and ethylmercury are thought to be very similar [67, 68]. Indeed, several studies have demonstrated that methylmercury increases intracellular ROS production, depletes intracellular glutathione, and acts on the mitochondria to depolarize the mitochondrial membrane potential and induce apoptosis [21, 69, 70].

In conclusion, we have determined that a subgroup of AD LCLs exhibits abnormal mitochondrial respiratory function at baseline and increased vulnerability to mitochondrial dysfunction when exposed to the environmental toxin, ethylmercury. This subgroup of AD LCLs has previously been shown to exhibit increased mitochondrial susceptibility to ROS, suggesting that these cells may be inherently vulnerable to a wide variety of oxidative insults. Pretreatment of this subgroup with NAC improved mitochondrial function at baseline and decreased the loss of mitochondrial reserve capacity in response to ethylmercury. Our data suggest that the abnormal mitochondrial function and increased susceptibility to ethylmercury in the AD-A LCLs may be related to impaired glutathione-mediated antioxidant capacity and chronic oxidative stress, since NAC pretreatment, which could improve glutathione status, appears to partially correct the atypical mitochondrial function in the AD-A LCLs and protect the cells against the toxic effects of ethylmercury. Other prooxidant environmental toxicants associated with ASD such as pesticides and polychlorinated biphenyls (PCBs) should be tested to determine whether these autism LCLs exhibit hypersensitivity to a wide range of prooxidant environmental toxicants as our findings support the notion that a subset of individuals with autism may be vulnerable to environmental influences with detrimental effects on development through mitochondrial dysfunction.

## Supplementary Material

The supplementary material contains the detailed results and figures for AD-N subgroup, including the comparisons between the AD-N LCLs and matched control LCLs (Figure S1) and the comparisons between the AD-N LCLs with and without NAC pretreatment (Figure S2).

## Figures and Tables

**Table 1:** Lymphoblastoid cell line characteristics and pairing between AD and control cell lines. The list is organized by the two groups previously identified: AD-A and AD-N.

Pair number	Autism Lymphoblastoid cell lines				Control Lymphoblastoid cell lines		
	Cell ID	Source	Age (y)	Subgroup	Cell ID	Source	Age (y)
1	03C14441	NIMH	7	AD-A	GM17255	Coriell	6
2	1165302	AGRE	13	AD-A	GM11626	Coriell	13
3	01C08594	NIMH	7	AD-A	GM05909	Coriell	28
4	01C08495	NIMH	4	AD-A	06C52389	NIMH	18
5	02C09713	NIMH	7	AD-A	GM11973	Coriell	7
6	02C10054	NIMH	6	AD-N	06C53370	NIMH	37
7	04C26296	NIMH	10	AD-N	05C49729	NIMH	36
8	00C04757	NIMH	10	AD-N	GM10153	Coriell	10
9	05C38988	NIMH	12	AD-N	GM16007	Coriell	12
10	03C15992	NIMH	5	AD-N	GM18054	Coriell	5
11	1267302	AGRE	10	AD-N	GM14643	Coriell	25
12	02C10618	NIMH	7	AD-N	GM05049	Coriell	22
13	02C09650	NIMH	7	AD-N	05C51773	NIMH	18
14	04C27439	NIMH	7	AD-N	04C27915	NIMH	30
15	01C08022	NIMH	5	AD-N	GM09380	Coriell	6
16	03C17237	NIMH	10	AD-N	05C49729	NIMH	36

NIMH: National Institutes of Mental Health (Bethesda, MD, USA).

AGRE: Autism Genetic Resource Exchange (Los Angeles, CA, USA).

Coriell: Coriell Cell Repository (Camden, NJ, USA).
