# Supplementary material for: Increased Susceptibility to Ethylmercury-Induced Mitochondrial Dysfunction in a Subset of Autism Lymphoblastoid Cell Lines
Source: J Toxicol. 2015 Jan 21;2015:573701. doi: 10.1155/2015/573701 (PMC4320799; doi:10.1155/2015/573701)
Supplement: Supplementary file 1 — The supplementary material contains the detailed results and figures for AD-N subgroup, including the comparisons between the AD-N LCLs and matched control LCLs (Figure S1) and the comparisons between the AD-N LCLs with and without NAC pretreatment (Figure S2). [file 573701.f1.pdf]

Supplementary Results

AD-N v control LCLs.

ATP-linked respiration was slightly higher in the AD-N as compared to control LCLs [$F(1,442)=18.96$, $p<0.0001$]. While ATP-linked respiration decreased as mercury increased [$F(6,59)=32.64$, $p<0.0001$], this decrease was not different between groups (Figure S1A). ATP-linked respiration was found to be significantly lower than baseline at 0.125μM [$t(59)=2.13$, $p<0.05$], 0.25μM [$t(59)=2.14$, $p<0.05$], 0.5μM [$t(59)=4.60$, $p<0.0001$], 1.25μM [$t(59)=7.58$, $p<0.0001$] and 2.5μM [$t(59)=11.76$, $p<0.0001$].

Overall proton leak respiration was slightly but significantly higher in the AD-N as compared to the control LCLs [$F(1,442)=38.16$, $p<0.0001$] (Figure S1B). Proton leak respiration significantly changed as mercury increased [$F(6,59)=2.96$, $p<0.05$], but this change was not significantly different between the two groups. This change in proton leak respiration with increasing mercury was due to a significantly lower proton leak respiration at 2.5μM mercury as compared to baseline [$t(59)=3.03$, $p<0.01$].

Maximal respiratory capacity was slightly higher in the AD-N as compared to control LCLs [$F(1,442)=7.13$, $p<0.01$]. While maximal respiratory capacity decreased as mercury increased [$F(6,59)=41.70$, $p<0.0001$], this decreased was not different between groups (Figure S1C). Maximal respiratory capacity was found to be significantly lower than baseline at 0.125μM [$t(59)=2.01$, $p<0.05$], 0.25μM [$t(59)=2.49$, $p<0.05$], 0.5μM [$t(59)=5.69$, $p<0.0001$], 1.25μM [$t(59)=8.79$, $p<0.0001$] and 2.5μM [$t(59)=12.22$, $p<0.0001$].

Overall reserve capacity was not different between the LCL groups (Figure S1D). Reserve capacity decreased as mercury increased [$F(6,59)=37.18$, $p<0.0001$], but this change was not significantly different between the two LCL groups. Reserve capacity was significantly lower

than baseline at 0.25μM [t(59)=2.44, p<0.05], 0.5μM [t(59)=5.64, p<0.0001], 1.25μM [t(59)=8.36, p<0.0001] and 2.5μM [t(59)=11.23, p<0.0001].

AD-N LCLs: NAC Pretreatment vs No Pretreatment

As seen in Figure S2A, pretreatment with NAC slightly but significantly increased ATP-linked respiration [F(1,505)=23.00,p<0.0001]. ATP-linked respiration for both the pretreated and the non-pretreated AD-N LCLs decreased as mercury increased [F(6,59)=33.65,p<0.0001] but this decrease was not different across the two LCL groups. ATP-linked respiration was significantly lower than baseline at 0.125μM [t(59)=2.07, p<0.05], 0.25μM [t(59)=3.13, p<0.01], 0.5μM [t(59)=4.00, p<0.001], 1.25μM [t(59)=7.72, p<0.0001] and 2.5μM [t(59)=11.98, p<0.0001].

Pretreatment with NAC slightly but significantly increased proton leak respiration [F(1,505)=10.74, p=0.001] (Figure S2B). Proton leak respiration for both the pretreated and the non-pretreated AD-N LCLs changed as mercury increased [F(6,59)=3.36, p<0.01] but this decrease was not different across the two LCL groups. Proton leak respiration was significantly lower than baseline at 2.5μM [t(59)=3.33, p<0.01].

Pretreatment with NAC slightly but significantly increased maximal respiratory capacity [F(1,505)=5.20,p<0.05] (Figure S2C). Maximal respiratory capacity for both the pretreated and the non-pretreated AD-N LCLs decreased as mercury increased [F(6,59)=53.11,p<0.0001] but this decrease was not different across the two LCL groups. Maximum respiratory capacity was significantly lower than baseline at 0.25μM [t(59)=3.57, p<0.001], 0.5μM [t(59)=5.92, p<0.0001], 1.25μM [t(59)=10.02, p<0.0001] and 2.5μM [t(59)=13.92, p<0.0001].

Pretreatment with NAC did not influence overall reserve capacity (Figure S2D). Reserve capacity for both the pretreated and the non-pretreated AD-N LCLs decreased as mercury increased [F(6,59)=47.42, p<0.0001] but this decrease was not different across the two LCL groups. Reserve capacity was significantly lower than baseline at 0.25μM [t(59)=3.33, p=0.001],

0.5μM [t(59)=5.94, p<0.0001], 1.25μM [t(59)=9.50, p<0.0001] and 2.5μM [t(59)=12.78, p<0.0001].

Supplementary Figures

Figure S1

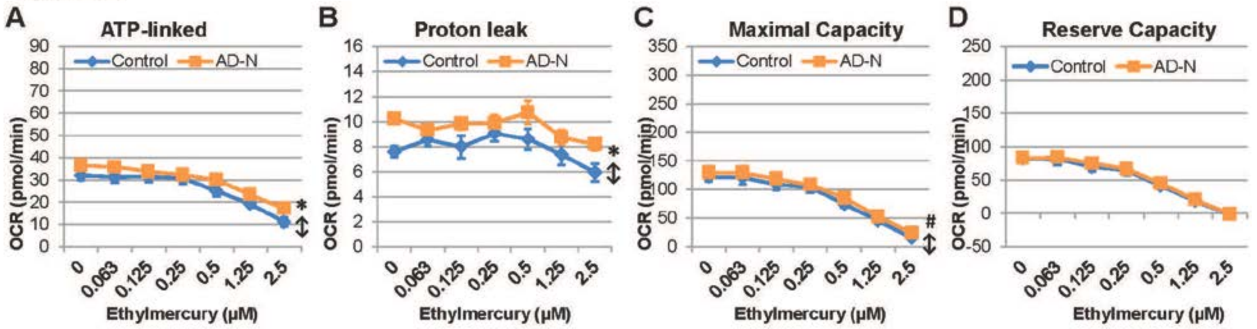

Figure S2

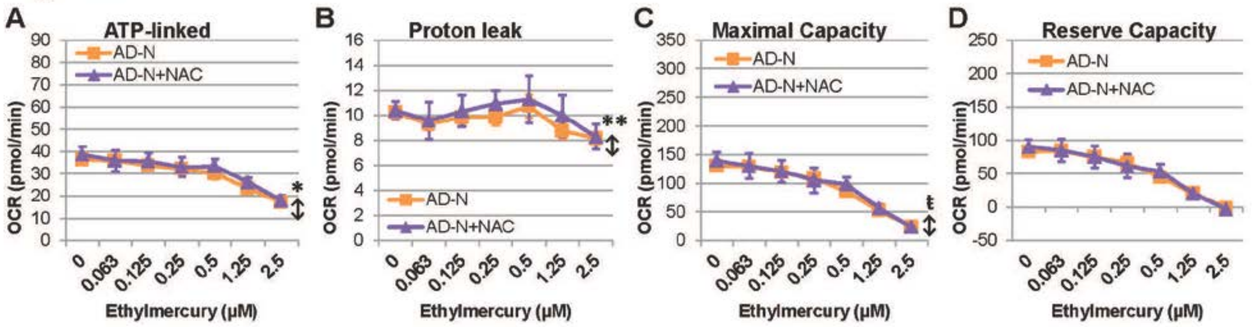

Supplementary Figure Legends

Figure S1. Mitochondrial respiratory parameters and responses to mercury in the AD-N LCL subgroup. Overall, the AD-N subgroup demonstrates similar mitochondrial responses as the control LCLs. (A) ATP-linked respiration (B) proton leak respiration and (C) maximal respiratory capacity are overall slightly but significantly higher in the AD-N LCLs as compared to controls. *p≤0.0001; # p≤ 0.01; ↨indicates an overall statistical difference between LCL groups when differences at individual concentrations of mercury are not significant.

Figure S2. Mitochondrial respiratory parameters and responses to mercury in the AD-N LCL subgroup following 48 hour pretreatment with 1mM N acetyl-cysteine (NAC). Overall, changes in mitochondrial function with increased mercury seen in the AD-N subgroup are not drastically different following NAC pretreatment. (A) ATP-linked respiration, (B) proton leak respiration and (C) maximum respiratory capacity are slightly but significantly higher in the NAC pretreated group as compared to the not pretreated group. *p≤0.0001; **p≤0.001; ŧ p≤0.05; ↨indicates an overall statistical difference between LCL groups when differences at individual concentrations of mercury are not significant.
